# Visualisation of γH2AX Foci Caused by Heavy Ion Particle Traversal; Distinction between Core Track versus Non-Track Damage

**DOI:** 10.1371/journal.pone.0070107

**Published:** 2013-08-14

**Authors:** Nakako Izumi Nakajima, Holly Brunton, Ritsuko Watanabe, Amruta Shrikhande, Ryoichi Hirayama, Naruhiro Matsufuji, Akira Fujimori, Takeshi Murakami, Ryuichi Okayasu, Penny Jeggo, Atsushi Shibata

**Affiliations:** 1 Research Center for Charged Particle Therapy and International Open Laboratory, National Institute of Radiological Sciences, Chiba, Japan; 2 Genome Damage and Stability Centre, University of Sussex, Brighton, East Sussex, United Kingdom; 3 Japan Atomic Energy Agency, Tokai, Japan; National Taiwan University, Taiwan

## Abstract

Heavy particle irradiation produces complex DNA double strand breaks (DSBs) which can arise from primary ionisation events within the particle trajectory. Additionally, secondary electrons, termed delta-electrons, which have a range of distributions can create low linear energy transfer (LET) damage within but also distant from the track. DNA damage by delta-electrons distant from the track has not previously been carefully characterised. Using imaging with deconvolution, we show that at 8 hours after exposure to Fe (∼200 keV/µm) ions, γH2AX foci forming at DSBs within the particle track are large and encompass multiple smaller and closely localised foci, which we designate as clustered γH2AX foci. These foci are repaired with slow kinetics by DNA non-homologous end-joining (NHEJ) in G1 phase with the magnitude of complexity diminishing with time. These clustered foci (containing 10 or more individual foci) represent a signature of DSBs caused by high LET heavy particle radiation. We also identified simple γH2AX foci distant from the track, which resemble those arising after X-ray exposure, which we attribute to low LET delta-electron induced DSBs. They are rapidly repaired by NHEJ. Clustered γH2AX foci induced by heavy particle radiation cause prolonged checkpoint arrest compared to simple γH2AX foci following X-irradiation. However, mitotic entry was observed when ∼10 clustered foci remain. Thus, cells can progress into mitosis with multiple clusters of DSBs following the traversal of a heavy particle.

## Introduction

Radiotherapy represents a widely employed treatment for cancer with around fifty per cent of cancer patients receiving radiotherapy [1]. X-rays have been employed as the major radiation source for radiotherapy for many years. However, recent advances have provided evidence that alternative types of radiation, including protons and heavy ion can be efficiently and beneficially utilised. The major benefit of charged heavy particle irradiation is that a large dose can be delivered to the tumour whilst minimising damage to the surrounding healthy tissue [2]. The nature of the DNA damage caused by heavy ions, the type of radiation studied here, is distinct to that induced by X or γ-rays. However, whilst our knowledge of the energy deposition and the cellular mechanisms employed following exposure to X or γ-rays has increased dramatically in recent years, our understanding of the damage caused by heavy ion irradiation is more fragmentary. Indeed, the precise nature of the DNA damage induced, the mechanism of repair and how such damage activates other DNA damage responses (DDR) such as cell cycle checkpoint arrest are unclear. Although studies have shown that the checkpoint machinery has limitations after X or γ-ray exposure, how these pathways are regulated after heavy ion exposure has not been examined [3]. Addressing these questions is essential if heavy ions are to be widely utilised for therapeutic benefit not least for considering whether secondary malignancies can arise following therapy. Additionally, understanding the impact of heavy ions, particularly Fe ions, is important to evaluate the risks associated with space exploration [4].

The radiation quality is defined by its linear energy transfer (LET), which determines the spatial density of energy deposition events, and is determined by the particle type, charge and energy. Heavy particle irradiation, such as Fe or Carbon ions, has high LET which causes intense deposition of energy within nanometre volumes. DNA double strand breaks (DSBs) induced by low LET radiation such as X or γ-rays can have associated base damage or single strand breaks (SSBs) but closely localised DSBs arise infrequently whereas high LET radiation frequently induce highly complex DSBs, in which multiple DSBs can arise within one to two helical turns (10–20 nm) from a single particle track [5]. The close localisation of DSBs within a few helical turns, which can arise after high LET radiation, is encompassed within the category of complex DSBs [5]. An additional consideration is that heavy ions deposit their energy within tracks created by the particle's traversal through the cell [6]. Thus, the DSBs can be closely localised along a track. A further consideration of relevance here, is the generation of secondary electrons, termed delta rays, which arise from the initial ionisation event. These secondary electrons can have a range of energies and hence distribution, and can traverse in multiple directions over substantial distances [7], [8], [9]. The majority of delta rays do not traverse large distances and give rise to DSBs closely localised at the position at which they are generated, i.e. within the particle track. However, high energy delta electrons can traverse substantial distances (of the order of µm) from the particle track (see subsequent discussion). Indeed, they are predicted to be able to traverse the diameter of a cell. Thus, it is predicted that such high energy delta electrons could cause low LET damage similar to that of X-rays away from the particle track [10]. Physical models have provided strong evidence that delta electrons cause DSBs after high LET irradiation and studies have shown that the indirect action of water-derived radicals can contribute to cell killing after high LET radiation [11], [12], [13]. However, the characterisation of DNA damage distant from the particle track has not been rigorously examined in cells.

Our understanding of the DNA damage response (DDR) induced by DNA DSBs has advanced enormously in the past few years. Most specifically, it has become realised that chromatin modifications in the vicinity of a DSB extend over several hundred and even thousand base pairs [14], [15]. The phosphorylation of H2AX, a variant form of the histone H2A, by ATM and DNA-PK, has proved to be an invaluable approach to examine DSB induction and repair [16]. Phosphorylated H2AX, termed γH2AX, can be monitored as discrete foci and their numbers after X- or γ-ray irradiation represent a good monitor of DSB formation and their rate of loss provides a monitor of DSB repair in non-replicating G0/G1 cells [17]. Several studies have shown that γH2AX foci after heavy particle irradiation are larger and more diffuse than those arising after X-ray exposure, and are deposited in tracks [18], [19]. Given that phosphorylated H2AX from a simple X-ray induced DSB covers greater than one hundred base pairs, the analysis of γH2AX foci does not have the capacity to resolve complex DSBs (localised damage arising within 10–20 nucleotides) that arise following high LET radiation [20]. Nonetheless, larger and persistent foci have been observed after such radiation [18], [21]. One goal was to determine whether the γH2AX foci arising after high and low LET irradiation could be distinguished. As a component of this goal, we aimed to determine whether delta electron induced damage arising at sites away from the particle tracks could be observed and distinguished from the damage arising within the particle tracks. For this analysis, Fe ion particles were employed since they were predicted to induce significant high energy delta electron damage [7], [22]. We also assessed whether the slowly repaired larger γH2AX foci arising after heavy particle irradiation can act as a more complete barrier to checkpoint arrest compared to X-ray induced DSBs. Addressing these questions is essential to allow subsequent analysis such as differences in repair and processing of distinct classes of DSBs. We observed that the large, diffuse γH2AX foci detectable by 2D imaging microscopy within the particle tracks encompass multiple smaller foci, which can be distinguished by 3D imaging and deconvolution. We have defined these as clustered foci. We additionally provide a detailed assessment of DNA damage induced by delta electrons distant from the particle track. We show that these γH2AX foci are similar to those induced by X or γ-rays and are distinct in nature to the clustered foci formed within the particle tracks. We also demonstrate that clustered γH2AX foci have enhanced capacity to signal to the checkpoint machinery but nonetheless that cells with large clustered γH2AX foci can enter mitosis.

## Materials and Methods

### Cell culture and irradiation

48BR (Wild type) primary, 1BR (WT) and 2BN (XLF defective) hTERT human fibroblasts were cultured in DMEM supplemented with 15% fetal calf serum, 100 U/ml penicillin and 100 µg/ml streptomycin at 37°C in a humidified mixture of 95% air and 5% CO_2_. To obtain G0/G1 confluency, cells were seeded onto Nunc chamber glass slides ∼7 days prior to irradiation. Exposures to carbon (290 MeV/n, LET 70 keV/µm) and iron (500 MeV/n, LET 200 keV/µm) beams were performed at the Heavy Ion Medical Accelerator (HIMAC) facility of the National Institute of Radiological Sciences (NIRS), Chiba, Japan. Chamber slides were set up in a horizontal (approximately 5 degrees) or vertical position. The beam characteristics, biological irradiation procedures and dosimetry using HIMAC have been described previously [23], [24]. The spatial distribution of the fluence was monitored to have a variation less than +/−10% within well sizes (0.8×0.8 cm^2^) on both the X and Y directions. The particle fluence traversing a cell after 1 Gy horizontal irradiation was estimated to be ∼1.1, considering an LET value of 200 keV/µm for the Fe particles and the average diameter of the cell nucleus (12.67 µm diameter×2.8 µm nuclear depth). We also utilised Fe ions with a condition described as a “a pencil beam”, in which no shaping devices were placed in the beam course, in order to minimise the contamination of fragment particles. The use of a “pencil beam” has been described previously [24], [25]. The purity of Fe ions was estimated to be around 90% in the present case. X-ray irradiation was performed at 200 kVp and 20 mA with Aluminium (0.5 mm)-Copper (0.5 mm) filters (Shimadzu, TITAN-320, NIRS). Dose rates were set at 0.5 Gy/min for X-rays. 10 µM ATM inhibitor, KU55933 (Merck Chemicals, Darmstadt, Germany) and/or 10 µM DNA-PKcs inhibitor, NU7441 (Merck Chemicals, Darmstadt, Germany) was added 30 min prior to irradiation. These concentrations have been shown in previous studies to specifically inhibit ATM or DNA-PK, respectively [26]. X-rays cause an increase in dose for cells grown on glass cover slips relative to plastic surfaces, which was taken into account in the dose assessment as described previously [27].

### Immunofluorescence staining

Cells were washed in cold PBS and fixed for 10 min in 4% w/v paraformaldehyde. Cells were then permeabilised for 2 min in 0.2% v/v Triton X-100 (Sigma-Aldrich, Japan) in PBS, and washed twice in PBS. Antibodies were diluted with 4% w/v BSA in PBS. Cells were incubated with mouse anti-γH2AX antibody (Millipore, Billerica USA) and CENP-F (Abcam, Tokyo, Japan) or pSer10-histone H3 (Millipore, Billerica USA) for 1 h at 37°C, washed three times in PBS and incubated with FITC-conjugated rabbit anti-mouse IgG antibody (Sigma-Aldrich, Japan) for 1 h at room temperature. Slides were incubated in PBS containing DAPI (4′,6-diamidino-2-phenylindole) for 5 min to stain the DNA and mounted using Vectashield (Vector lab, CA, USA).

### Cluster γH2AX foci analysis by DeltaVision microscopy using softWoRx deconvolution

Microscopic images were captured with an Applied Precision® DeltaVision® RT Olympus IX70 deconvolution microscope using a 100× objective. Since the depth of 48BR and XLF cells is approximately <2–3 µm, 20 slices within 4 µm were taken with Z-series stacks. Images taken by the DeltaVision were deconvoluted and processed using softWoRx. 20 deconvoluted images were stacked into a single-layer image to analyse γH2AX foci on photoshop 5.5 (Adobe, San Jose, CA). To enumerate the number of tiny γH2AX foci within a foci cluster, we selected a 1.5×1.5 µm^2^ area, which is sufficient to distinguish clusters which are 0.5 µm apart from neighbouring clusters. Very few clusters appeared greater than this. Foci length was measured by ImageJ v1.35p following image processing. Foci width using 3D analysis was measured by IMARIS software (Bitplane AG, Zurich, Swizerland).

### γH2AX foci scoring

Cluster foci scoring was carried out blindly with >30 cells/experiment using a Zeiss Axioplan or Olympus BX51 microscope. Unless stated otherwise all foci analysis represents the mean and SD of 3 experiments. For most experiments, results were consolidated by 2–3 people undertaking the scoring. For delta electron analysis, γH2AX foci were enumerated at non-track regions excluding a zone of 2 µm from the track. To examine γH2AX foci in G2 phase in cycling cells, 4 µM aphidicolin (APH) was added immediately after irradiation. APH treatment blocks the replicative polymerases and hence progression from S to G2 phase. APH induces pan-nuclear γH2AX signal in S phase [28]. Cells were stained with γH2AX and CENP-F (G2 marker). APH does not affect DSB repair including γH2AX foci formation, NHEJ or homologous recombination in cells derived from G2 phase cells. Full controls for the use of APH have been previously undertaken [28], [29].

### G2/M checkpoint analysis

For G2/M checkpoint analysis, exponentially growing cells were seeded on glass coverslips 48 h before irradiation. Following irradiation, 4 µM APH was added to prevent progression from S to G2/M phase. Arrested S phase progression during the time course of analysis was confirmed by FACS [28], [29]. Cells were stained with pSer10-histone H3 (Millipore, Billerica USA) and DAPI. pSer10-histone H3-positive and condensed chromatin cells were counted as mitotic cells.

### Statistical analysis

All data were derived from 3–4 independent experiments except where stated. Statistical significance was determined using Student's two-tailed t test or Mann-Whitney U test by SigmaPlot 12.0. *P<0.05, **P<0.01, ***P<0.001. Analysis was carried out between control versus others unless stated.

### Simulated track structure analysis

Details of the simulation method have been described elsewhere [9]. Briefly, Monte Carlo simulation code TRACION was used to simulate the track structure. The simulated track (energy deposition points) was superimposed on a simple cell nucleus model for scoring DNA damage. In the model used, linear-B-form DNA segment (150 bp length) is randomly distributed to achieve the DNA concentration in the cell nucleus. The DNA is assumed to be surrounded by water. A DNA concentration of 13.4 Mbp/µm^3^ was used here, which was obtained by our experimental observation. The induction of DNA strand breaks by direct and indirect pathways is simulated as followings. The energy deposition events on the DNA molecule are assumed to directly induce strand breaks when >10 eV energy is deposited in the sugar-phosphate moiety. The energy deposition events in water are converted to initial water radicals such as OH radicals. These radicals diffuse and react with DNA. When a OH radical reacts with a sugar-phosphate moiety, a strand break is assumed to be induced with a probability of 0.13. A DSB is scored when two SSBs exist on opposite strands within 10 bp.

## Results

### Examination of γH2AX foci formed within tracks generated by Fe ion irradiation

**Figure 1:**
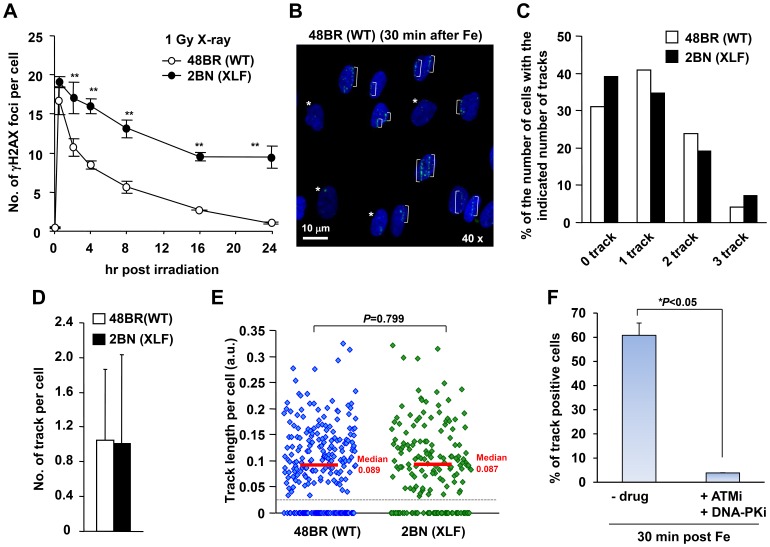
Variation in the length and number of tracks between individual flat fibroblast cells. (A) γH2AX foci in 48BR (WT) primary and 2BN (XLF) hTERT cells were enumerated from 0.5–24 h post 1 Gy X-rays. Foci were scored in 2D using a Zeiss Axioplan microscope. (B) 48BR (WT) cells were fixed at 30 min following 1 Gy Fe ions and stained with γH2AX and DAPI. Asterisks represent non-track cells. (C) Distribution in the percentage of γH2AX tracks following Fe irradiation in B. (D) The average number of tracks per cell following 1 Gy Fe is shown. The predicted fluence of particles traversing the nuclease after 1 Gy horizontal Fe irradiation was estimated to be ∼1.1 with a Poisson distribution predicting and ∼70% cells receiving a particle track (see Materials and Methods). (E) Scatter plots of track length per cell following 1 Gy Fe irradiation are shown in 48BR (WT) and 2BN (XLF) G0/G1 cells. Tracks whose length are <0.025 were excluded from the data, since they were indistinguishable from delta-electron induced foci. ∼250 cells were examined in each analysis (C–E). (F) Clustered γH2AX foci within the tracks is ATM/DNA-PK dependent, indicating that they are DSBs. 48BR (WT) cells were fixed at 30 min post Fe irradiation with/without ATM plus DNA-PK inhibitor. To examine the percentage of track positive cells, >200 cells were scored. Error bars represent the standard deviations (SD) from 2 experiments. The analysis was performed by DeltaVision microscope without deconvolution (B–F). Note that although the cells were exposed to 1 Gy Fe ions, the dose to individual cells can differ due to differing number of particles traversing the cell. In the ensuing analysis, we examine cells which have a single particle traversal.

Initially, we examined γH2AX foci within tracks generated by the traversal of Fe ion particles. To assess the ability of the γH2AX foci to be repaired by DNA non-homologous end-joining (NHEJ), we examined 48BR (WT) primary and 2BN (XLF-defective) hTERT fibroblasts. XLF is a core component of the ligation complex of the NHEJ machinery; in the absence of XLF, DSB repair following exposure to X-rays is severely impaired (Figure 1A) [26], [30]. We used G0/G1 cells following contact inhibition to preclude repair events in S or G2 phase; the presence of >95% G1 cells was verified by FACS (data not shown). Fibroblasts were exposed to 1 Gy Fe ions in a horizontal direction (at 5 degrees to the track), which yielded the maximum number of cells with a single particle track (Figure 1B–C). We observed a variable length and number of tracks between the individual flat fibroblast cells likely because the particles can centrally or peripherally traverse the cells and because the plane of individual cells differs substantially. Thus, although the dose received by the population of cells is 1 Gy, the dose to any individual cell within the population can be distinct. However, despite significant variation between individual cells, the average number of tracks and track length per cell was similar between control and XLF cells (Figure 1D–E). Thus, horizontal irradiation induces DSBs equally in control and XLF cells despite significant variation between individual cells.

To verify that the foci within the tracks represent DSBs, we examined their formation following ATM plus DNA-PKcs inhibitor treatment since they represent the kinases promoting H2AX phosphorylation specifically at DSBs in G0/G1 phase cells (Figure 1F and S1A) [31]. The treatment of these inhibitors completely abolished γH2AX foci formation within the particle tracks, demonstrating that they represent DSBs.

Using either 48BR or XLF cells, we observed dense γH2AX within the particle tracks from 30 min to 2 h (data not shown). Discrete foci in 48BR cells became evident by 2 and 8 h post irradiation. Discrete foci appeared to resolve more slowly in XLF cells but there was variation between cells making the difference difficult to quantify. In all experiments by 8 h post irradiation, defined foci were visible in both 48BR and XLF cells. Therefore, we examined the larger defined foci that were evident in particle tracks from 8 h onwards. Initially we focused our attention on γH2AX foci that were present in an obvious particle track. At 8 h post exposure, there were more foci remaining in XLF cells than in 48BR cells but they resolved over time in both 48BR and XLF deficient cells with a rate that was not dramatically different (Figure S1B).

### Identification of clustered γH2AX foci using enhanced resolution microscopy

**Figure 2:**
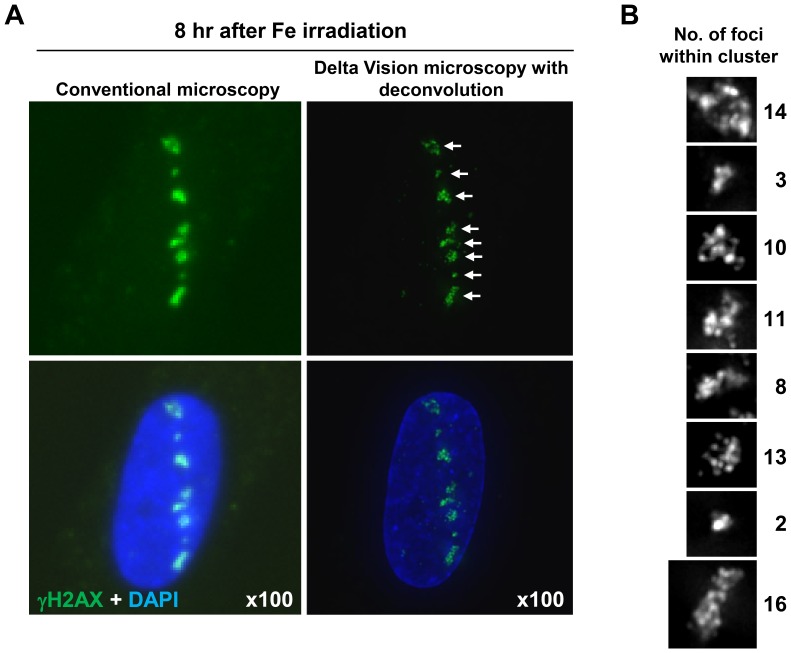
High resolution microscope analysis revealed clustered γH2AX foci formation within the tracks following Fe irradiation. (A, B) Clustered γH2AX foci formation in 48BR (WT) primary G0/G1 cells is visualised using deconvolution. Images were captured with an Applied Precision DeltaVision RT Olympus IX70 microscope with deconvolution (right). The image resolution using the DeltaVision is compared with Zeiss Axioplan microscope without deonvolution (left). Enlarged individual clustered foci are shown with grey scale in B.

Using a conventional microscope and 2D imaging (without deconvolution), the foci present at 8 h post Fe ion exposure were large and diffuse (Figure 2A, left). To gain further insight into the nature of these foci, we examined their structure using an Applied Precision DeltaVision RT Olympus IX70 deconvolution microscope. This 3D imaging with deconvolution revealed that the larger γH2AX foci (hereafter called clustered γH2AX foci) contain multiple smaller foci that can be visually distinguished at 8 h post 1 Gy Fe ions (in cells with a single particle track) (Figure 2A, right: N.B. the image is shown in 2D after stacking of 3D images sections). We analysed the complexity of these γH2AX foci by enumerating the number of individual foci within a cluster using the DeltaVision microscope coupled with 3D sectioning and deconvolution (scoring examples are shown in Figure 2B) (see Materials and Methods for further details).

**Figure 3:**
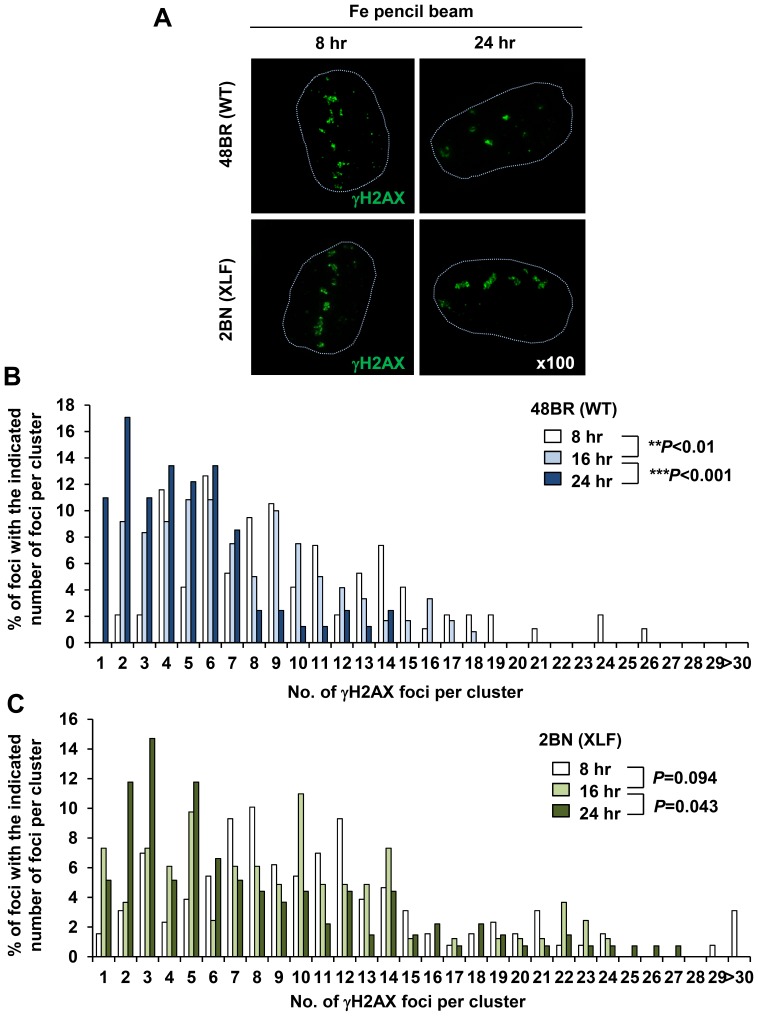
Clustered γH2AX foci arising within the particle tracks represent a signature of high LET particle radiation and are repaired slowly by NHEJ in G1. (A) 48BR (WT) primary and 2BN (XLF) hTERT G0/G1 cells were irradiated in a horizontal direction with 1 Gy Fe irradiation. Images are taken using the DeltaVision microscope followed by deconvolution. Representative images at 8 and 24 h post Fe irradiation are shown. The nucleus outline is drawn with a dashed line from the DAPI staining. (B, C) Percentage of individual foci within a cluster was analysed from >100 individual clusters at each time point. Similar results were obtained in two independent experiments. Cells forming a single γH2AX track of length >8 µm and width >1 µm were analysed.

We measured the distribution of the complexity of γH2AX foci in 48BR and XLF cells at 8–24 h following Fe ion exposure (Figure 3A). In the experiments described above, we utilised an Fe ion beam that was estimated to encompass some contaminating ion particles that arise during ion travel. To avoid consideration of such contaminating particles in these experiments (and most importantly for the experiments in the next section), we used an Fe ion beam with >90% Fe particles (hereafter called Fe pencil beam; see Materials and Methods) [24], [25]. This analysis revealed that in 48BR cells, the complexity of the γH2AX foci (i.e. the number of foci encompassed within a single cluster) diminished from 8 to 16 to 24 h post exposure (Figure 3B). Significantly, in XLF cells, there was a slightly greater level of complexity at 8 h compared to 48BR cells and by 24 h there was little change in the magnitude of complexity, such that by 24 h, there was substantially greater complexity than in 48BR cells (Figure 3C). The fact that the level of clustered foci did not show any increase in XLF cells from 8 to 24 h indicates that they do not represent coalescing of foci.

**Figure 4:**
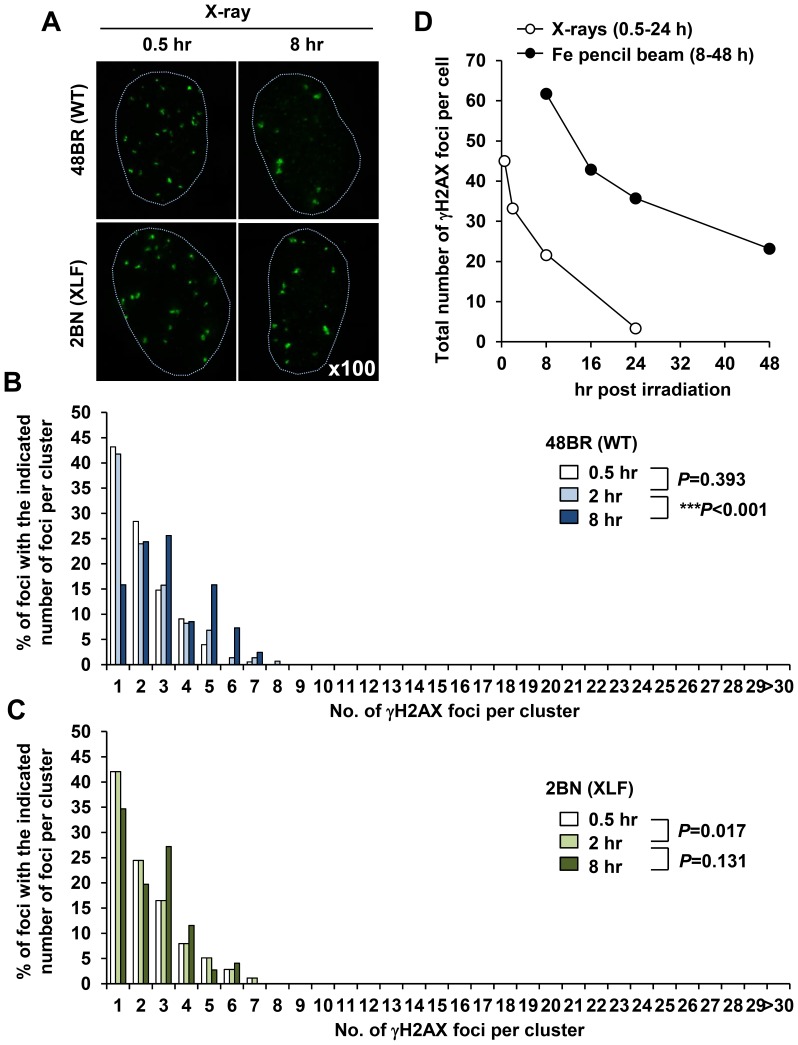
X-irradiation induces ‘Simple’ γH2AX foci. (A) 48BR (WT) primary and 2BN (XLF) hTERT G0/G1 cells were irradiated with 1 Gy X-rays and stained with γH2AX and DAPI. Images are taken using the DeltaVision microscope followed by deconvolution. (B, C) Distribution of foci numbers in clusters was analysed from >100 individual clusters at each time point. (D) Repair kinetics following 1 Gy Fe ions is slower than that after 1 Gy X-rays. To examine repair kinetics, the total number of γH2AX foci within all the clusters per cell were enumerated from the cluster analysis in Figure 3B and 4B. Similar results were obtained in two independent experiments.

To verify that clustered γH2AX foci which form within the tracks are a signature of heavy ion particle radiation, we conducted similar analysis following X-irradiation. Since the DSB repair kinetics following X-rays is faster than Fe ion irradiation, we performed clustered γH2AX foci analysis from 0.5–8 h post 1 Gy X-rays (Figure 4A). Importantly, we did not observe any highly clustered γH2AX foci formation as detected following Fe irradiation although minor (and very much smaller) clustered foci with ∼3–5 individual foci could be identified (Figure 4B). Interestingly, we also observed a small increase in the percentage of foci with 4–7 individual foci at 8 h in 48BR cells suggesting that these larger clustered foci may be slowly repair in comparison to the simpler foci (Figure 4B; we consider the possible origin of these foci in the discussion section). We stress, however, that the clustered foci arising after X-irradiation are substantially smaller than those observed after Fe ion irradiation raising the possibility that their origin is distinct. Additionally, similar to the Fe ion irradiation results, XLF cells did not exhibit any increase in the overall number of clustered foci with time consistent with the notion that clusters do not represent merging of foci with time (Figure 4C). It is notable also that whereas the number of single centred foci decreased significantly in 48BR cells by 8 h post IR, the decrease was much less in XLF cells. Next, to investigate the DSB repair kinetics of individual foci, we plotted the total number of individual γH2AX foci per cell following exposure to Fe ions. Importantly, Fe ion induced individual γH2AX foci are repaired slowly compared to those of X-ray induced foci (Figure 4D).

In summary, using deconvolution microscopy, we show that the large γH2AX foci visible by low resolution microscopy at later times following Fe ion irradiation represent clustered γH2AX foci which contain individual discrete foci. These clustered foci arise within the Fe particle track and are repaired slowly and represent a form of lesion that provides a signature for high-LET damage since they are distinct to the foci arising after X-rays.

### Identification of small non-clustered γH2AX foci distinct from the particle track

**Figure 5:**
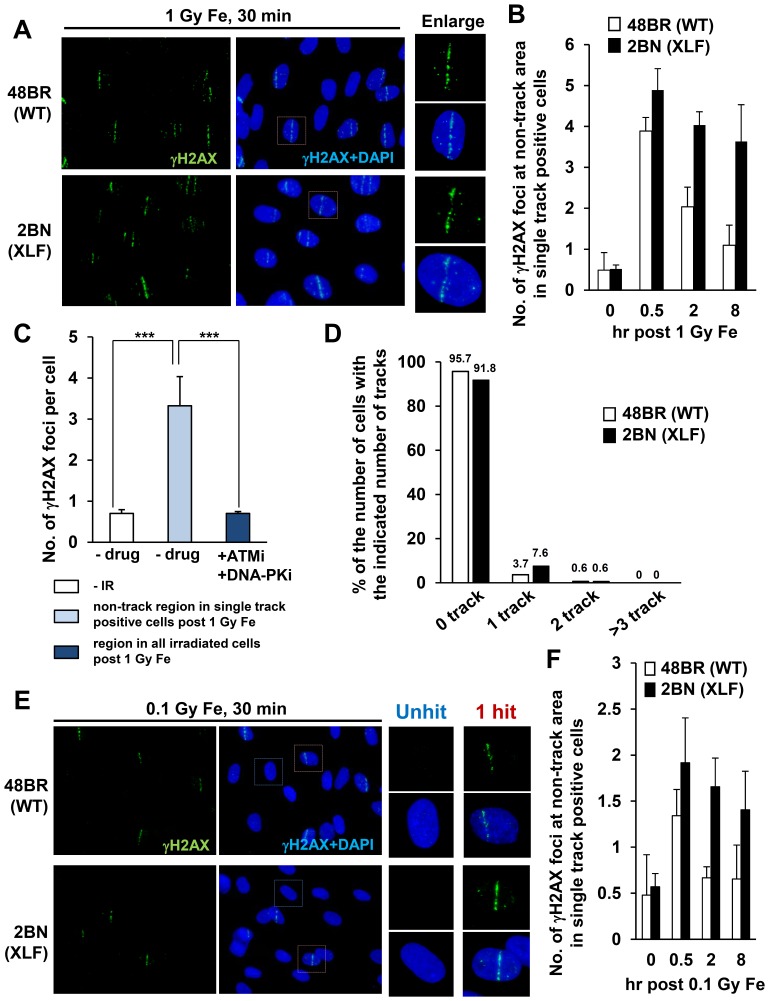
Generation of “simple” γH2AX foci at distances away from the particle track and they are repaired rapidly by NHEJ. (A) Representative image of γH2AX foci formation at non-track regions. 48BR (WT) primary and 2BN (XLF) hTERT G0/G1 cells were irradiated with 1 Gy pencil Fe ions and stained with γH2AX and DAPI. (B) γH2AX foci at non-track regions in cells which have a single track per nucleus were enumerated following 1 Gy Fe ion irradiation. (C) To investigate whether non-track induced γH2AX foci formation is due to DSBs, the number of γH2AX foci at non-track regions was enumerated with/without ATM plus DNA-PK inhibitor. Since ATM/DNA-PK inhibitor treated cells do not form γH2AX tracks, foci number was enumerated without any bias in these cells. (D) Distribution in the percentage of γH2AX tracks following 0.1 Gy Fe irradiation. (E, F) γH2AX foci at non-track regions with a single track per nucleus was examined following 0.1 Gy Fe irradiation. A region which is located greater than 2 µm from the track was excluded from the analysis (B and F). Images were taken by Olympus BX51 microscope without deconvolution (A and E). γH2AX foci were analysed using Olympus BX51 or Zeiss Axioplan microscope by 2D (B, C, D and F). Cells forming a single γH2AX track of length >8 µm and width >1 µm were analysed in B and F.

During our analysis of Fe ion irradiation given in a horizontal direction, we observed γH2AX foci located at distances away from the particle track (Figure 5A). These γH2AX foci were particularly evident in XLF cells at early times post IR. We considered whether these could represent damage caused by high energy delta electrons. Firstly, we enumerated the number of γH2AX foci at non-track regions selecting cells which have a single track per nucleus and focusing on those located greater than 2 µm from the track (we chose a wide margin to be certain that they are distinct from damage arising within the tracks). We observed ∼4 γH2AX foci at non-track regions per single track positive cell, whereas the number of background foci without DNA damage is <1 in both 48BR and XLF G0/G1 cells (Figure 5B). Significantly, XLF cells showed a substantially reduced rate of loss of these non-track γH2AX foci, consistent with the notion that they represent DSBs and require NHEJ for their repair (Figure 5B). An elevated number of these foci were observed at 30 min post exposure in XLF compared to 48BR cells, a feature also observed following exposure to X-rays, which we suggest is due to ongoing repair in 48BR cells during the 30 min post exposure time (Figure 1A and 5B). It was difficult to examine later times post exposure due to difficulty in distinguishing track from non-track damage. To further consolidate that these foci represent DSBs, we examined their dependence on ATM/DNA-PKcs by the addition of their specific inhibitors. Since γH2AX foci do not form in tracks following addition of these inhibitors (Figure 1F), we enumerated the number of γH2AX foci in the entire population and observed complete loss of foci formation (Figure 5C). To substantiate that these foci do not represent the remains of partial tracks, potentially arising, for example, by traversal of a particle through a small part of the cell, we reduced the radiation dose from 1 to 0.1 Gy which resulted in only 1 per 10–20 cells being traversed by a particle (Figure 5D). Importantly, we observed Fe induced γH2AX foci within non-track regions as classified above in those cells harbouring a track although the number was reduced relative to that observed after 1 Gy (Figure 5E–F, also see discussion section).

**Figure 6:**
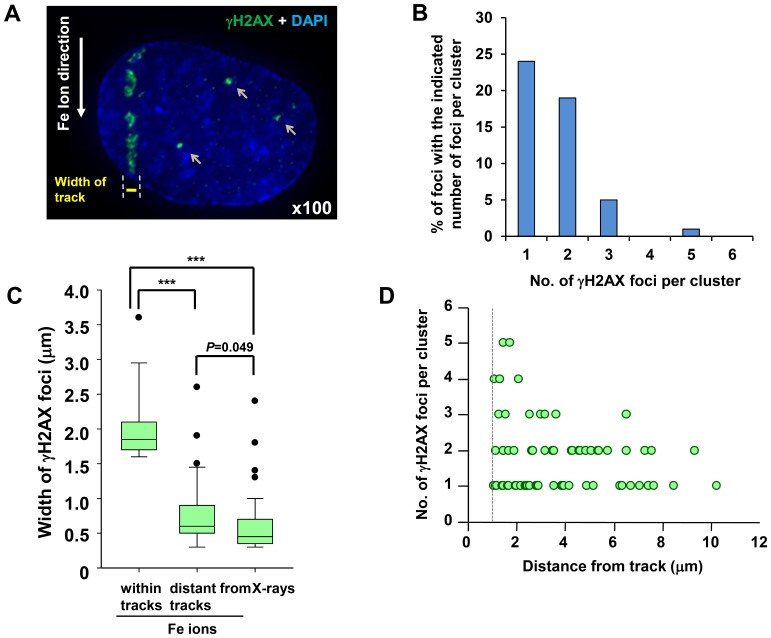
γH2AX foci at sites distinct to the tracks showing less clustered damage. (A) Typical image of DSB formation outside of the particle track following Fe ion irradiation. 48BR (WT) primary G0/G1 cells were irradiated with 1 Gy Fe irradiation, fixed at 30 min, and stained with γH2AX and DAPI. (B) Percentage of individual foci within a cluster at non-track regions was analysed from >100 individual foci. 48BR (WT) cells were irradiated and fixed at 30 min post 1 Gy Fe irradiation. (C) The width of clustered γH2AX foci post Fe irradiation within the particles tracks, at non-track regions following Fe irradiation or following X-irradiation. >50 foci were analysed in each sample. The diameter of clustered γH2AX foci after Fe ions is ∼3–4 fold greater than the width of γH2AX foci arising after X-rays, whilst the foci diameter of non-track γH2AX foci is similar to that arising following X-rays. The width of foci is measured by IMARIS in 3D using images taken by the DeltaVision microscope. (D) Foci complexity is negatively correlated with distance from the particle track. γH2AX foci at non-track regions in 48BR cells were analysed following 1 Gy Fe irradiation. Cluster analysis was performed as described in Figure 3–4. To allow the analysis of γH2AX foci close to the particle track, we used a distance of 1 µm to distinguish non-track from track damage and carried out the analysis at 30 min exposure to minimise the movement of γH2AX foci within tracks. Images were taken by DeltaVision microscope followed by deconvolution and analysed (A–D). Cells forming a single γH2AX track of length >8 µm and width >1 µm were analysed in B and C.

We also assessed the complexity of the non-track γH2AX foci following exposure to 1 Gy Fe ions. Importantly, foci arising >2 µm distance from the track predominantly had a level of complexity similar to those arising after X-rays (Figure 6A–B). A similar distribution was observed in 0.1 Gy irradiated cells (data not shown). Additionally, we measured the width of the γH2AX foci generated by Fe ions versus X-rays at 30 min post irradiation using 3D imaging software (IMARIS) without stacking, i.e. the values represent the actual width in 3D. We observed a 2–3 fold lower width size for foci induced by X-rays compared to Fe ions (Figure 6C). We also observed that the width of γH2AX foci arising at sites distant from the tracks were similar to those arising from X-ray exposure (Figure 6C). We carried out this analysis at 30 min to avoid difficulties caused by the movement of the γH2AX foci within tracks. Finally, we also estimated the complexity of non-track γH2AX following deconvolution microscopy as a function of distance from the particle track (Figure 6D). This analysis was done at 30 min post irradiation and we included foci >1 µm from the particle track to encompass a broader range of delta electron induced damage. This analysis revealed a diminished complexity of γH2AX foci with distance from the particle track. Collectively, we report the generation of “simple” γH2AX foci at distances away from the particle track. These foci are less complex than those forming within the particle track, consistent with the notion that they could represent DSBs arising from high energy delta electron damage.

### Track Structure and simulated spatial distribution of DSBs

**Figure 7:**
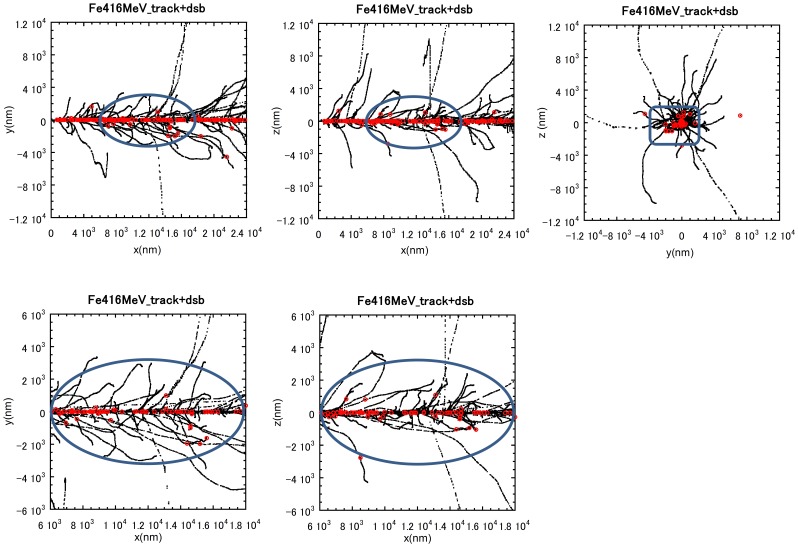
Track structure simulation and spatial distribution of DSBs arising from an Fe ion track. A Simulation of the structure of damage arising from a single Fe ion particle traversal. The black marks and the red marks represent the tracks of Fe ions and the position of DSBs, respectively. The blue ellipsoid shape shows the size of the nucleus. The slighter wider band of the damage within the tracks observed in these experiments is likely explained by DNA movement after radiation exposure. The number of electrons generated decreases as the electron energy decreases. For 416 MeV/n Fe ions, ∼90% of electrons generated are estimated to have energies less than 100 eV. The range of such low-energy electrons is less than a few nm. The majority of other electrons have a range from a nm to 5 µm. The highest energy electrons with a range of a few hundred µm can also arise but at a low frequency (<0.1%).

To further assess our experimental data, we carried out a computer simulation of the track structure and the predicted distribution of DSBs along the track path. An example of a simulated track segment of 416 MeV/n Fe ion with predicted DSB formation is shown in Figure 7. The particles were generated with an energy of 500 MeV/n and were estimated to have an energy of 416 MeV/n at the contact point with the cells. Thus, 416 MeV/n has been used in this simulation. The representative size of the nucleus is shown by the blue ellipsoidal area. This analysis shows that DSBs are intensively formed along the ion projectile. From this simulation, the total number of DSBs formed within a cell's nuclear volume is 84; the number of DSBs within a radial distance of 1 µm from the ion projectile is 78 whilst 6 DSBs are predicted to be localised outside of a 1 µm radius. These findings are consistent with our observations where the majority of DNA damage lies within 1 µm of the particle trajectory whilst we observed 2–5 γH2AX foci located beyond 2 µm of the track damage.

### Cells with clustered γH2AX foci can enter mitosis despite prolonged checkpoint arrest following exposure to Fe ions

**Figure 8:**
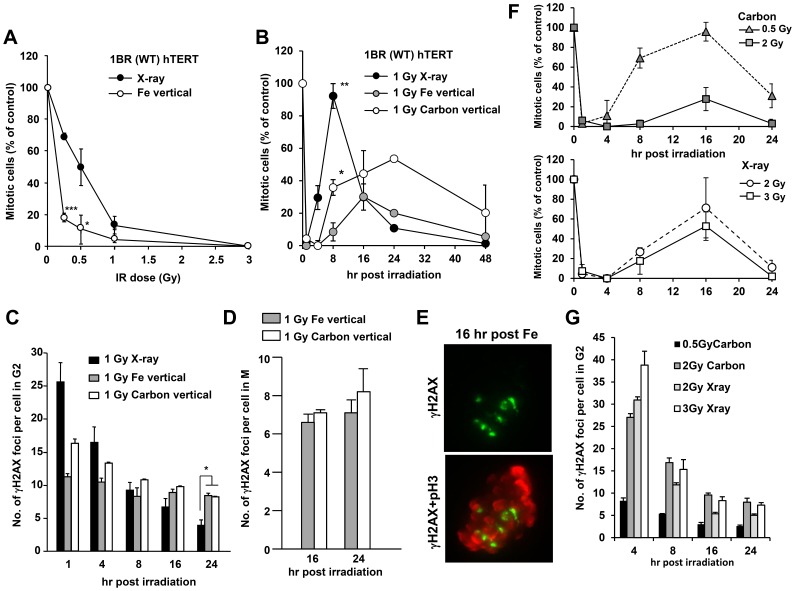
Cells with clustered γH2AX foci can enter mitosis despite prolonged checkpoint arrest following exposure to Fe ion irradiation. A) Mitotic entry was examined in 1BR (WT) hTERT cells at 1 h post X-rays or vertical Fe irradiation. Cells, >400, were scored for mitotic index (MI). Mitotic cells were identified by p-histone H3 Ser10 and cellular morphology by DAPI. Results represent the MI relative to untreated cells. 1BR hTERT cells shows similar G2/M checkpoint responses to that in 48BR primary cells [40]. The size and number of γH2AX foci clusters between 48BR primary and 1BR hTERT cells were similar (data not shown). (B) The time of mitotic entry was examined following exposure to 1 Gy X-rays, Fe or Carbon ion irradiation. (C) The number of clustered γH2AX foci in G2 cells was enumerated by 2D analysis using normal microscopy following 1 Gy X-rays, Fe or Carbon ion irradiation. G2 cells were identified by CENP-F staining. (D) Since irradiated G2 cells restart cell cycle progression from 16 h post heavy ion irradiation, the number of DSBs in mitotic cells at 16 and 24 post IR was examined by scoring γH2AX foci in p-histone H3 Ser10 positive M phase cells. (E) Typical image of γH2AX foci in metaphase 1BR hTERT cells were taken by DeltaVision 3D imaging. (F) The maintenance of checkpoint arrest was examined following 0.5, 2 Gy Carbon or 2, 3 Gy X-rays. (G) γH2AX foci in G2 cells were enumerated following 2 Gy Carbon or X-rays. Mitotic index and γH2AX foci in G2 or M cells were examined by 2D analysis using Olympus BX51 or Zeiss Axioplan microscope.

We have previously shown that following X-irradiation, 15–20 γH2AX foci are required to initiate and maintain cell cycle checkpoint arrest [32]. We next aimed to compare the number of clustered γH2AX foci induced by Fe ion irradiation required to initiate or maintain checkpoint arrest. We examined checkpoint arrest after vertical irradiation because the distribution of damage to individual cells after horizontal irradiation was too broad for detailed analysis after different doses. Additionally, we did not utilise the pencil beam irradiation because we observed a greater variation in particle tracks induced per cell compared to that obtained with the normal beam and because we considered that a small fraction of contaminating particles would be of less impact compared to the analysis above. The vertical irradiation also allowed manipulation of the number of DSBs induced per cell with increasing dose with less variation in the extent of DNA damage between cells (Figure S2A–B). Our findings suggested that vertically irradiated cells initiate checkpoint arrest at lower doses compared to X-irradiated cells (Figure 8A). Thus, checkpoint arrest was observed after 0.25 Gy carbon or Fe irradiation, which induces 3–5 vertical tracks, whereas checkpoint arrest is inefficiently activated after 0.25 Gy X-rays [32]. The checkpoint arrest following exposure to Fe ion was ATM-dependent, since arrest was abolished following ATM inhibition (data not shown).

Next we evaluated the number of clustered γH2AX foci required to maintain checkpoint arrest and whether cells were able to enter mitosis with such clustered lesions. Following exposure to 1 Gy Fe ions given vertically, we monitored the mitotic index (MI) up to 48 h post irradiation. To analyse the timing of checkpoint release of irradiated G2 cells but not irradiated G1/S cells, aphidicolin, a DNA replication polymerase inhibitor, was added to prevent S phase cells entering into G2/M phase during analysis [29]. Exposure to 1 Gy X-rays resulted in checkpoint release around 4 h post IR and nearly full release by 8 h (Figure 8B). In contrast, exposure to 1 Gy Fe ion irradiation resulted in cells entering mitosis from 16 to 48 h post IR. Next, we enumerated the number of clustered γH2AX foci present in irradiated G2 cells at the time of checkpoint release (Figure 8C). We observed approximately 10 clustered foci in cells from 8 to 24 h post 1 Gy Fe ion irradiation. In contrast, the G2/M checkpoint is released when ∼15 γH2AX foci remain after X-irradiation [32]. We also examined whether γH2AX foci could be identified in the mitotic cells arising during checkpoint release. Although the number of γH2AX foci scored was lower than in G2 cells, foci were clearly visible in mitotic cells (Figure 8D). A representative image of a metaphase cell containing clustered γH2AX foci is shown in Figure 8E. We also attempted to estimate the number of clustered foci present following vertical irradiation based on our data obtained from horizontal irradiation. Considering the depth of a fibroblast cell (estimated for 48BR to be ∼2.8 µm from 3D DeltaVision analysis) and the number of clustered foci formed following horizontal irradiation, we estimated that only ∼1–2 clustered foci per track may arise following vertical irradiation. Consistent with this, we could readily identify clustered foci using deconvolution microscopy after vertical irradiation (data not shown). The number of individual foci per vertical track was similar to that observed in a single clustered foci generated by horizontal irradiation at 24 h post exposure, consistent with the notion that, due to the length of the vertical tracks only ∼1 clustered foci arise per track. Based on our analysis, we observed that each clustered foci may harbour on average ∼5 smaller foci at 24 h post exposure. Since checkpoint release occurred when approximately 10 clustered foci remained following vertical irradiation, we estimated that 50 smaller foci in G2 were detectable at the time of checkpoint release. Clearly, however, this analysis has inherent in exactitude. Finally, to substantiate the notion that checkpoint arrest after heavy ions is released when fewer γH2AX remain compared to exposure to X-irradiation, we carried out checkpoint analysis using carbon ion irradiation which is exploited clinically for heavy ion therapy. The timing of checkpoint release after carbon ions was slightly faster than that observed after Fe ions which likely correlates with the distinct damage complexity between these two types of heavy particle irradiations, i.e. LET between Carbon (70 keV/µm) and iron (200 keV/µm) (Figure 8B). Importantly, however, the number of clustered γH2AX foci present at the time of checkpoint release following carbon ion exposure was ∼10, e.g. 8 h post 1 Gy; 16 h post 2 Gy, similar to that obtained after 1 Gy Fe ions (Figure 8F–G). In contrast, ∼15 foci are present when release is initiated following X-ray exposure, e.g. 4 h post 1 Gy; 8 h post 2 Gy (Figure 8F–G).

These results demonstrate two important findings. Firstly, they show that cells can progress into M phase even with clustered DSBs following exposure to heavy ion irradiation and support our previously reported findings that the G2/M checkpoint is insensitive. Secondly, they show that the magnitude of signalling from a clustered focus is likely greater than from a “simple” focus arising after X-irradiation since checkpoint arrest is initiated with lower doses and release commences when ∼10 clustered γH2AX foci remain.

## Discussion

High LET radiation can be encountered from a range of exposures including flying, exposure to radon, and perhaps most significantly from the increased usage of charged particle radiotherapy. Given the increased longevity post radiotherapy, it is important to understand the impact of charged particles in cellular systems and whether lesions that can induce carcinogenesis might persist in surviving cells. In this study, we have exploited recent advances in our understanding of the DNA damage response to DSB formation and used deconvolution microscopy to gain insight into the nature of DSBs induced by charged particles and their cellular impact.

### Clustered γH2AX foci are a signature of high LET heavy particle irradiation

At early times post irradiation, intense γH2AX foci formation occurs within the particle tracks, which is often difficult to resolve into defined foci. It appeared that this dense γH2AX formation took longer to resolve in XLF-deficient cells compared to control cells but variation between cells makes it difficult to quantify. Nonetheless, these findings suggest that there is a form of DNA damage within the particle tracks, which is rapidly repaired. At early times post irradiation, it was difficult to reliably quantify defined γH2AX foci within the particle tracks. Thus, we commenced analysis of damage within the particle tracks from 8 h onwards. At 8 h post Fe ion exposure in control cells, we observed large γH2AX foci, which, by deconvolution microscopy, could be shown to encompass multiple smaller foci. These foci contain up to ∼30 individual foci while those forming after X-rays rarely have <7 individual foci/cluster. Thus, these highly clustered foci represent a hall mark of high LET radiation. The presence of larger and persistent γH2AX foci after high LET damage and the dependency on NHEJ for repair has been reported previously, although detailed analysis of their complexity has not been hitherto described [5], [18], [19], [21], [33], [34], [35]. Perhaps surprisingly, we also observed some foci clustering following exposure to X-rays although such clustered foci were much smaller, less complex and distinct in nature to that observed after high LET irradiation. Interestingly, the complexity of the clustered foci following Fe ions diminished with time (from 8 to 24 h) in control cells, suggesting that the individual foci within a cluster are repaired separately gradually generating less clustered foci. The question raised is what such clustered γH2AX foci represent. Physical track structure modelling has strongly suggested that clustered DSBs arising from a single high LET ionisation event form within a few helical turns [20]. It is, however, highly unlikely that these individual foci represent distinct clustered DSBs arising in this manner since the magnitude of foci at a single DSB extends over multiple kilo-base pairs. One possible explanation for the clustered foci is that they arise in regions where there could be uneven foci expansion due to diminished phosphorylation by distinct chromatin status. Studies in yeast have shown that γH2AX expansion does not arise evenly throughout the genome and that specific regions, such as transcriptionally active regions, are refractory to phosphorylation [36]. However, this explanation might predict that individual foci within a cluster would be lost in unison when the causal DSB undergoes repair. Another possibility is that they represent DSBs forming within regions of heterochromatin that migrate together to the heterochromatin periphery [37]. Whilst we consider these reasonable explanations for the clustering observed after X-irradiation, these two possibilities are less appealing as an explanation for the clustered foci observed after Fe ion irradiation. A third explanation is that the closely localised γH2AX foci represent closely localised individual DSBs, a possibility strengthened by the finding that the magnitude of clustering diminishes slowly with time in control cells but not in XLF cells, suggesting that each γH2AX foci is repaired independently. A recent study, which also observed clustered γH2AX foci after Fe ion irradiation, suggested that DSBs can localise to repair factories after radiation exposure [35] and this also represents an explanation for our findings. Although several explanations are possible, we tentatively suggest that they could represent closely located DSBs that arise from the significant level of closely located damage including delta electron induced DSBs, and that such close localisation impairs the rate of DSB repair. Although their precise origin is currently unclear, they represent an important biomarker of high LET irradiation. Another study recently visualised the recruitment of base excision repair proteins at huge 53BP1 foci (another DSB marker) following exposure to Fe ion demonstrating that complex lesions including highly dense base damages at DSB sites is another signature of heavy ions irradiation [5].

### Identification of non-track DNA damage arising from delta electrons

Biophysical modelling has predicted that delta electrons from secondary ionisation events can cause DSBs both within and distant to the particle track [11], [12]. We provide evidence for DSB induction by delta electrons by our analysis of DSBs arising at distances away from the particle track. Most importantly, we exploit XLF cells, which have impaired DSB repair, to visualise and identify these rapidly repaired DSBs. Our finding shows that DSBs arising at >2 microns from the particle track are X-ray like in size and magnitude of complexity. Further such DSBs are repaired rapidly in an XLF-dependent manner. To minimise the possibility that these DSBs could be due to multiple traversals of a particle track through a cell, we undertook a similar analysis following exposure to 0.1 Gy Fe ions, where only a minor fraction of the cells received a particle traversal. Although the number of delta electrons per cell was reduced compared to the analysis after 1 Gy Fe ions, we still observed evidence of DSBs distant from the particle tracks, most particularly in XLF cells, where they persist for longer times. The reduced number of X-ray like γH2AX foci observed at 0.1 Gy could be a consequence of delta electron damage arising in cells from their generation in the surrounding medium or neighbouring cells, which would be reduced at the lower dose. We think it unlikely that our analysis after 1 Gy included damage from particle tracks since the size, complexity and rate of repair of the foci formed was distinct to foci forming within the tracks, although this possibility cannot be eliminated. It is also important to note that we carried out these experiments using a pencil beam, which has >90% Fe ion particles within the beam. Further we carried out a simulation of track structure damage following Fe ion irradiation from which ∼6 DSBs were predicted to arise outside of the particle track in a cell having a single track traversal. This number is similar to our observations of 2–5 non-track DSBs. Collectively, we argue that these X-ray like DSBs are likely to represent those arising from high energy delta electrons.

### Cells can enter mitosis with clustered γH2AX foci

Finally, we examined whether the clustered γH2AX foci arising after high LET radiation would provide more efficient checkpoint arrest compared to those induced by X-rays. This analysis was based on our own and other studies that at low doses of X-rays, checkpoint arrest is not efficiently activated and that at higher doses, release occurs prior to the completion of DSB repair [32], [38]. We found that cells receiving 1 Gy Fe ions as vertical irradiation, although undergoing checkpoint arrest, were released from arrest even though a substantial number (around 10) of clustered γH2AX foci remained. Indeed, the mitotic cells showed clear evidence of harbouring γH2AX foci. Similar findings were also observed after exposure to a range of doses of carbon ions. For X-ray exposure we have previously, and here, observed that checkpoint release occurs when ∼15 γH2AX foci remain [32]. This analysis is important in considering whether cells with complex DSBs can progress through the cell cycle. Although p53 dependent G1/S checkpoint is a more sensitive checkpoint, the ability of cells with unrepaired DSBs to enter mitosis diminishes the opportunity for accurate DSB repair and raises the possibility for translocation formation [39]. The inefficient G2/M checkpoint has also been shown to contribute to chromosome breakage in mitotic cells after X-rays, leading to 1–2 chromosome breaks per cell in cells released from checkpoint arrest after X or γ-ray exposure [32]. It will be interesting to examine chromosome breakage in the cells released from checkpoint arrest after particle irradiation to examine whether the more complex γH2AX foci lead to enhanced chromosome breakage and/or aberration formation. After X-rays, we have shown that DSBs can be detected using premature chromosome condensation (PCC) in G2 cells at the time of checkpoint release and that ∼3 γH2AX foci correlate with 1 PCC break [32].

In summary, using 3D microscopy and deconvolution, we show that the large γH2AX foci formed after Fe ion irradiation encompass multiple, discrete smaller foci. These clustered foci are lost with slow kinetics and their complexity diminishes with time via an NHEJ-dependent process. Such foci, which can contain 10 or more individual foci, represent a signature of high LET radiation and are distinct to those induced by X-rays, which rarely contain more than 7 individual foci. Previous analysis has also observed large, persistent foci, with multiple γH2AX foci in close proximity, after high LET radiation [21]. We also demonstrate the presence of γH2AX foci at regions distant from the particle track that are similar to those arising after X-irradiation. We suggest that such DSBs that arise distinct from the tracks represent those arising from high energy delta electrons. Our findings suggest that the clustered γH2AX foci more efficiently activate checkpoint arrest compared to the smaller foci arising after X-irradiation. However, their efficiency in signalling is less efficient than anticipated from the estimated number of individual foci within a cluster. Importantly, however, we demonstrate that cells with up to 10 clustered γH2AX foci can progress into mitosis post exposure to Fe ions.

## Supporting Information

Figure S1 **H2AX phosphorylation following heavy ion irradiation is ATM/DNA-PK dependent.** (A) To verify that the foci within the tracks and at non-track regions represent DSBs, γH2AX foci formation was examined following ATM plus DNA-PK inhibitor treatment in G0/G1 phase 48BR cells. The drugs were added 30 min prior to irradiation and left until fixation. Quantification of the foci within the tracks and at non-track regions is shown in Figure 1F and 5C, respectively. Images are taken by the DeltaVision microscope without deconvolution. (B) The number of γH2AX foci in 48BR (WT) and 2BN (XLF) G0/G1 cells were enumerated from 8–72 h post 1 Gy Fe horizontal irradiation. Foci were scored by eye using a Zeiss Axioplan microscope.
(PDF)

Figure S2 **The number of DSBs induced per cell following vertical heavy ion irradiation has less variation in the extent of DNA damage between individual cells compared to horizontal irradiation.** (A) The number of γH2AX foci in 48BR (WT) primary G0/G1 cells were enumerated under a normal microscope at 30 min post 1 Gy X-rays, Fe and Carbon irradiations. (B) Scatter plot of γH2AX foci number post 1 Gy IR is shown. >60 cells per condition were examined. Less variation of γH2AX number between individual irradiated cells was observed following vertical Fe or Carbon irradiation compared to that observed following horizontal irradiation, although the variation is greater than that post X-rays.
(PDF)
