# Supplementary figures and images for: Visualisation of γH2AX Foci Caused by Heavy Ion Particle Traversal; Distinction between Core Track versus Non-Track Damage
Source: PLoS One. 2013 Aug 14;8(8):e70107. doi: 10.1371/journal.pone.0070107 (PMC3743843; doi:10.1371/journal.pone.0070107)

Figure S1

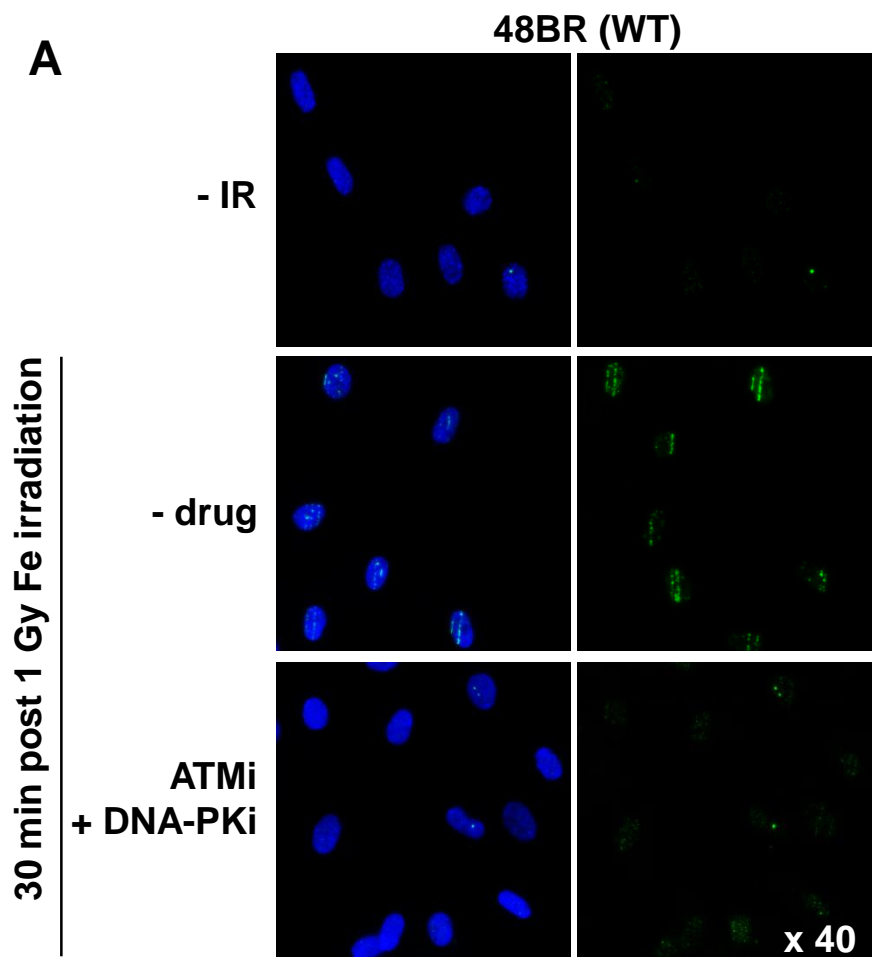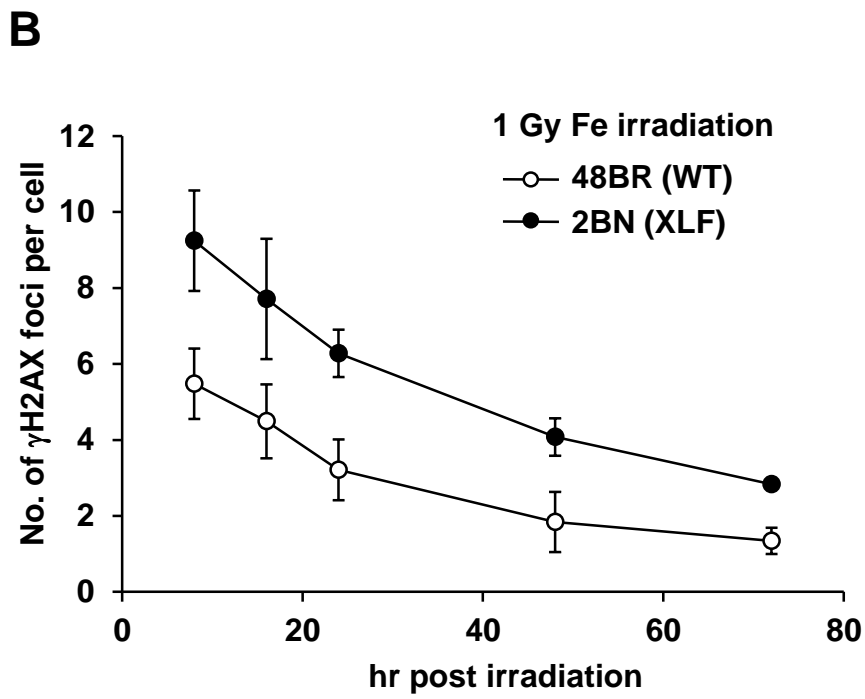

Supplement: Figure S1 — H2AX phosphorylation following heavy ion irradiation is ATM/DNA-PK dependent. (A) To verify that the foci within the tracks and at non-track regions represent DSBs, γH2AX foci formation was examined following ATM plus DNA-PK inhibitor treatment in G0/G1 phase 48BR cells. The drugs were added 30 min prior to irradiation and left until fixation. Quantification of the foci within the tracks and at non-track regions is shown in Figure 1F and 5C, respectively. Images are taken by the DeltaVision microscope without deconvolution. (B) The number of γH2AX foci in 48BR (WT) and 2BN (XLF) G0/G1 cells were enumerated from 8–72 h post 1 Gy Fe horizontal irradiation. Foci were scored by eye using a Zeiss Axioplan microscope. (PDF) [file pone.0070107.s001.pdf]

**Figure S2**

**A**

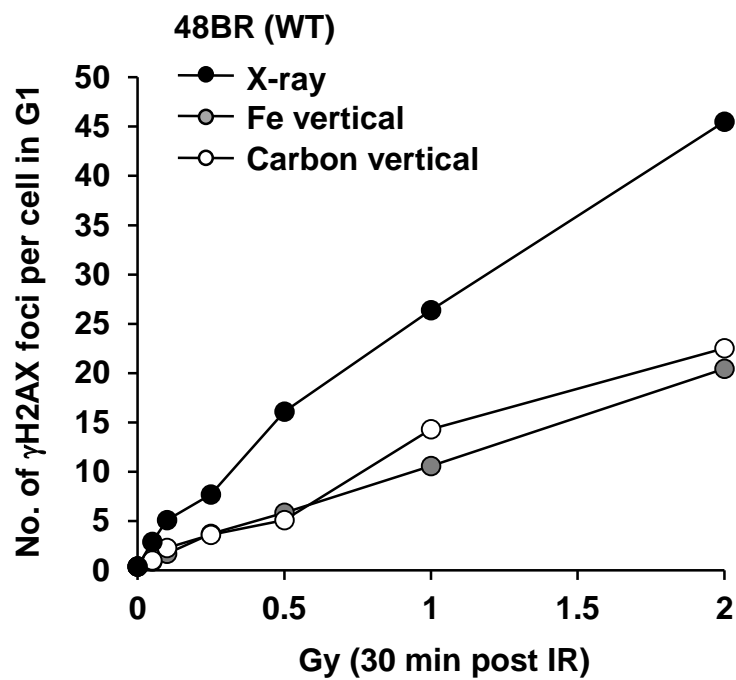

**B**

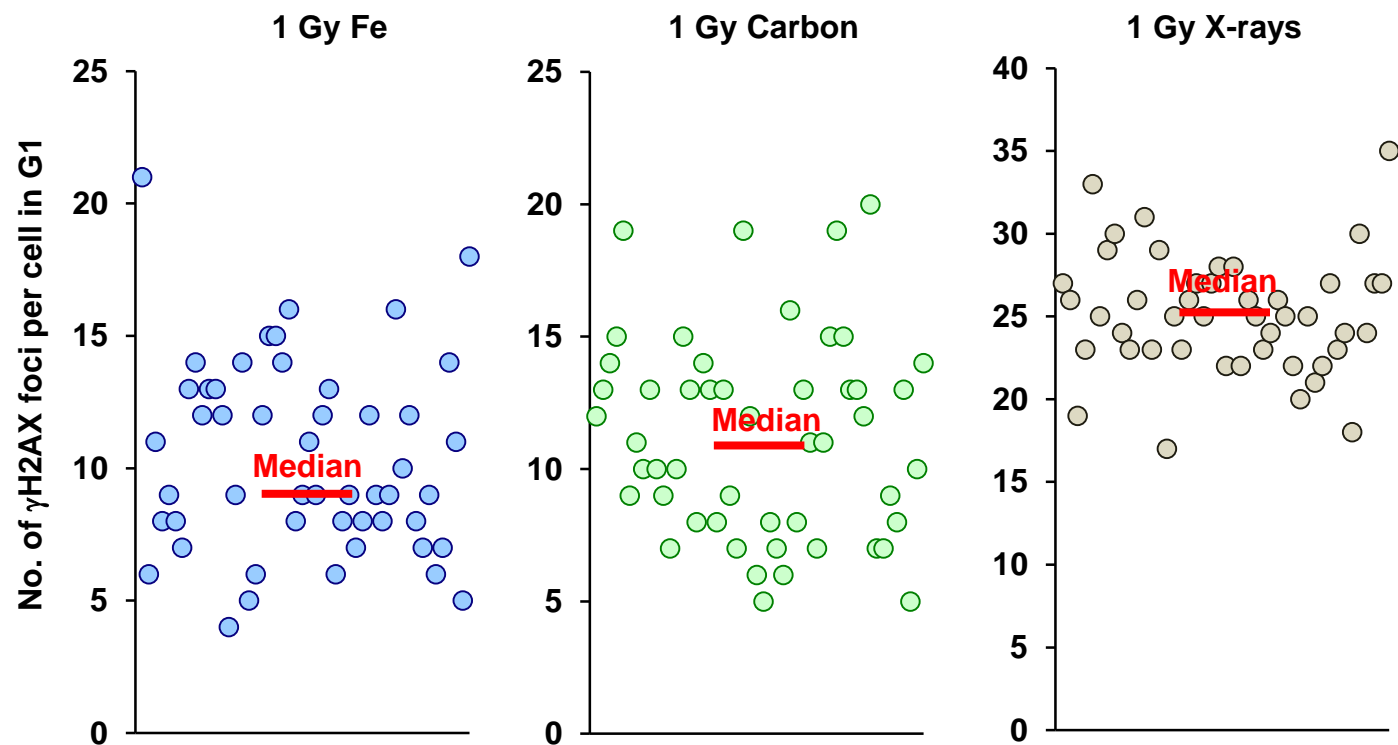

Supplement: Figure S2 — The number of DSBs induced per cell following vertical heavy ion irradiation has less variation in the extent of DNA damage between individual cells compared to horizontal irradiation. (A) The number of γH2AX foci in 48BR (WT) primary G0/G1 cells were enumerated under a normal microscope at 30 min post 1 Gy X-rays, Fe and Carbon irradiations. (B) Scatter plot of γH2AX foci number post 1 Gy IR is shown. >60 cells per condition were examined. Less variation of γH2AX number between individual irradiated cells was observed following vertical Fe or Carbon irradiation compared to that observed following horizontal irradiation, although the variation is greater than that post X-rays. (PDF) [file pone.0070107.s002.pdf]
